# Pathogenesis and signaling pathways related to iodine-refractory differentiated thyroid cancer

**DOI:** 10.3389/fendo.2023.1320044

**Published:** 2024-01-19

**Authors:** Simeng Zhao, Yuejia Zhao, Yongfu Zhao, Guangzhi Wang

**Affiliations:** Department of Thyroid Surgery, The Second Hospital of Dalian Medical University, Dalian, China

**Keywords:** RAIR-DTC, NIS, molecular mechanism, signaling pathway, targeted therapy

## Abstract

Thyroid cancer is the most common malignant neoplasm within the endocrine system and the field of head and neck surgery. Although the majority of thyroid cancers, more than 90%, are well-differentiated thyroid carcinomas with a favourable prognosis, the escalating incidence of this disease has contributed to an increasing number of patients with a propensity for recurrent disease, rapid disease progression, and poor or no response to conventional treatments. These clinical challenges are commonly attributed to alterations in key thyroid oncogenes or signaling pathways, thereby initiating tumour cell dedifferentiation events, accompanied by reduced or virtually absent expression of the sodium/iodine symporter (NIS). As a result, the disease evolves into iodine-refractory differentiated thyroid cancer (RAIR-DTC), an entity that is insensitive to conventional radioiodine therapy. Despite being classified as a differentiated thyroid cancer, RAIR-DTC has an extremely poor clinical prognosis, with a 10-year survival rate of less than 10%. Therefore, it is of paramount importance to comprehensively elucidate the underlying pathogenesis of RAIR-DTC and provide specific targeted interventions. As the pathogenic mechanisms of RAIR-DTC remain elusive, here we aim to review recent advances in understanding the pathogenesis of RAIR-DTC and provide valuable insights for the development of future molecularly targeted therapeutic approaches.

## 1 Introduction

Thyroid cancer, the most common malignancy of the endocrine system, has shown an alarming increase in incidence and mortality (1). Global data from 2018 revealed that there were approximately 567,233 new cases of thyroid cancer, accounting for 3.1% of all cancers studied. In addition, there were 41,071 deaths attributed to the disease, representing 0.4% of the total (2). Although the majority of thyroid cancers (over 90%) are well-differentiated papillary or follicular carcinomas with a favourable prognosis (3), approximately 10% of differentiated thyroid cancers develop distant metastases (4). More seriously, approximately one third of these metastatic cases exhibit an inability of thyroid cells to take up iodine, rendering them insensitive to radioiodine therapy and leading to the development of iodine-refractory differentiated thyroid cancer (RAIR-DTC). Current treatment options for this type of thyroid cancer, including surgical resection, radioiodine therapy and thyroid-stimulating hormone suppression, have limited or no therapeutic effect (5). Many of these cases are associated with mutations or degradation of the glycoprotein NIS in the plasma membrane of thyroid cells, such as mutations in the *SLC5A5* gene that encodes NIS. NIS plays a critical role in the uptake of iodine ions for the synthesis of thyroid hormones, thereby regulating the body’s metabolic activity from embryonic stages to senescence. Radioactive I^131^ treatment following thyroid cancer surgery also relies on NIS, perhaps with its long half-life of approximately 8 days, resulting in favourable clinical efficacy (6). These findings underscore the importance of NIS in various physiological functions and its indispensability in the treatment of thyroid cancer, particularly in cases resistant to radioiodine therapy. According to the 2015 American Thyroid Association Guidelines for the Diagnosis and Management of Thyroid Nodules and Differentiated Thyroid Cancer, RAIR-DTC is determined when one of the following indications is present: 1) complete loss of iodine uptake capacity for the lesion during the course of I^131^ treatment (for patients with imaging suggestive of metastasis but diagnostic I^131^ whole-body imaging confirming no iodine uptake, even if iodine uptake occurs on the whole-body image after subsequent I^131^ treatment, it may be difficult to fully benefit from I^131^ treatment because the tumour absorbed dose is insufficient to control the lesion, so this condition may also be considered RAIR-DTC); 2) an initial strong iodine uptake capacity of the lesion during I^131^ treatment, which gradually decreases over time; 3) heterogeneous iodine uptake within the lesion, with some cells showing uptake while others do not; 4) although the lesion has a strong iodine uptake capacity, the disease still progresses after high-dose I^131^ treatment (including lesion growth, appearance of new lesions, and sustained elevation of thyroglobulin levels). Literature reports indicate that the 10-year survival rate for thyroid cancer patients with loss of iodine uptake capacity is approximately 10%, with the 15-year survival rate dropping to approximately 6% (5). Although some FDA-approved RAIR-DTC-targeting drugs have been clinically developed, such as valprotectin (VCP)-containing inhibitors: clotrimazole and ibastine, they can selectively increase NIS expression activity, which in turn increases the sensitivity of patients to radioiodine therapy. However, due to the limitations of the drugs themselves and the complex and variable pathogenesis among individuals, there are still some patients who cannot benefit from this class of drugs (7). Therefore, the identification of key mutated genes, coupled with the development of targeted agents with definitive therapeutic efficacy, represents a promising approach to reduce the mortality rate of RAIR-DTC. In this paper, we will briefly review the molecular mechanisms associated with the onset and development of this disease, which may provide further selections for targeted therapies in RAIR-DTC.

## 2 Abnormal activation of the *RAS/BRAF/MEK/ERK* signaling pathway

The *RAS* cascade pathway predominantly controls essential cellular processes such as cell proliferation, motility, differentiation and apoptosis. However, extensive research has demonstrated aberrant activation of this pathway in various cancers, with mutations within its constituent elements serving as key drivers of tumourigenesis (8).

### 2.1 *RAS* mutations

*RAS*, a proto-oncogene, acts as a central “molecular switch” that regulates the entire signaling pathway. Once mutated, the entire signaling pathway deviates from its intended function and instead facilitates aberrant activities in certain tumour cells. *RAS* mutations have been identified in almost all types of thyroid cancer, particularly in cases characterised by distant metastasis and poorly differentiated tumour cells (9, 10). Notably, among the *RAS* genetic subtypes, *KRAS* has the highest susceptibility to missense mutations in various cancers, accounting for approximately 85%. However, in the context of thyroid cancer, *NRAS* is most susceptible to mutations, accounting for approximately 8%, while *KRAS* mutations account for only 1%. These observations highlight the tissue-specific nature of *RAS* subtype mutations. In addition, the *RAS* protein is susceptible to mutation at three hotspot codons, namely 12, 13 and 61 (11). Consistent with this, Cabanillas ME et al. reported the presence of *NRAS* ^Q61K^, *NRAS* ^G13D^ and *KRAS* ^G12V^ mutations in thyroid cancer patients (12). Another research team investigating guanine nucleotide releasing protein 3 (*RAS*GRP3), which encodes the *RAS* gene, found that mutations in *RAS*GRP3 promote thyroid cancer cell proliferation, migration and invasion, while reducing NIS expression and iodine uptake capacity. Whole-exome sequencing confirmed the high frequency of *RAS*GRP3 mutations in RAIR-DTC. Transfection of mutant *RAS*GRP3 (*RAS*GRP3-MUT) appeared to enhance the migratory and invasive abilities of thyroid cancer cells compared to transfection with wild-type *RAS*GRP3 (*RAS*GRP3-WT), accompanied by a decrease in iodine uptake capacity from 300×10^5^ to 150×10^5^. To elucidate the underlying mechanisms, the researchers treated *RAS*GRP3-MUT with a Phosphatidylinositol-3-kinase(PI3K)/protein kinase B(Akt) inhibitor, LY294002, and observed a decrease in Akt expression and invasive capacity of tumour cells, as well as an increase in NIS and TSHR expression in thyroid cells. Based on previous research findings, they postulated that the influence of *RASGRP3* mutation on the activity of thyroid cancer cells and NIS might be linked to the PI3K/Akt pathway (13). These findings suggest the potential existence of simultaneous mutations in multiple signaling pathways during the onset of this disease. Future investigations should consider the diverse etiology and multiple signaling pathways involved. This diagnostic perspective may offer valuable insights to guide future clinical studies on RAIR-DTC.

### 2.2 *BRAF* mutations

Another prominent activator within this pathway is the *RAF* family member, *BRAF*, which encodes a serine/threonine protein kinase (14). In particular, *BRAF* mutations are detected in approximately 30%-60% of thyroid cancers (15, 16), with papillary thyroid carcinomas showing mutation rates of up to 70% or more (17). More than 90% of these mutations correspond to *BRAF^V600E^*, which results from an adenine-thymine translocation at base 1799, replacing valine at position 600 with glutamate. This substitution leads to a 500-fold increase in *BRAF^V600E^* activity. Importantly, *BRAF^V600E^* activation is independent of upstream *RAS* signaling and can autonomously maintain the pathway at abnormally high levels of activity. Consequently, tumour cells with *BRAF^V600E^* mutations can thrive in a “favourable” microenvironment in the long term, enabling rapid growth, increased propensity for invasion and metastasis (18). In addition, Zhang Z et al. found that *BRAF^V600E^* down-regulates NIS gene expression by affecting the acetylation of NIS promoter histones. Initially, it was hypothesised that *BRAF^V600E^* decreases histone acetylation throughout the thyroid cell genome. However, the experimental results contradicted this assumption, as an increase in genome-wide acetylation was observed. Interestingly, despite the overall increase in histone acetylation, NIS gene expression decreased in thyroid cells. This led to the confirmation that *BRAF^V600E^* selectively downregulates histone acetylation within the critical region of the NIS gene promoter, thereby inhibiting NIS expression (19). In addition, Riesco-Eizaguirre G et al. observed that thyroid cells with *BRAF* mutations generally showed negative or weakly positive NIS staining, whereas thyroid cells without *BRAF* mutations showed normal NIS expression. This selective disruption of the transcriptional activity of the NIS promoter by *BRAF^V600E^* significantly affected NIS expression. Conversely, the transcriptional activities of the TPO, TSHR and Tg promoters were less affected (20). It can be concluded that the *BRAF^V600E^* variant may not affect thyroid function. Remarkably, the disruptive effect of *BRAF^V600E^* on NIS may not depend solely on the downstream MEK/ERK pathway, and the underlying mechanisms by which *BRAF^V600E^* reduces NIS activity require further investigation.

Compared to *RAS* and *RAF* mutations, isolated mutations in MEK and ERK leading to RAIR-DTC are relatively rare. Nevertheless, a limited number of studies have shown that MEK/ERK inhibitors can reduce the tumour burden of thyroid cells and increase NIS protein expression. For example, combined use of the MEK/ERK inhibitor PD98059 promotes NIS expression, resulting in increased iodine uptake. Notably, this effect is more pronounced when combined with the *BRAF^V600E^* inhibitor PLX4032. However, the use of the *BRAF^V600E^* inhibitor alone does not significantly increase NIS expression or iodine uptake in cells (21). Based on these findings, it is postulated that the high activity of *BRAF^V600E^* itself and the potent disruptive power of *BRAF^V600E^* on NIS transcriptional activity may render the use of a *BRAF^V600E^* inhibitor alone insufficient to reverse the downregulation of iodine uptake. Therefore, for RAIR-DTC patients with the *BRAF^V600E^* mutation, combination therapy consisting of a *BRAF^V600E^* inhibitor and a MEK/ERK inhibitor holds promise for synergistically enhancing NIS expression activity and restoring patients’ sensitivity to radioiodine therapy.

In conclusion, our findings suggest that irregular alterations in any element of the *RAS/RAF/MEK/ERK* signaling pathway may contribute to the development of RAIR-DTC. Therefore, directing treatment at this pathway shows substantial potential as a fundamental therapeutic strategy in the clinical context.

## 3 Activation of the phosphatidylinositol-3-kinase/protein kinase B signaling pathway

The PI3K/Akt pathway plays a critical role in regulating cellular processes, including cell proliferation, motility, and apoptosis. It is essential to understand the complex interactions of this pathway in controlling cellular processes to develop effective therapeutic strategies for malignancies. However, mutations in signaling molecules within this pathway or external influences can lead to the comprehensive regulation of diverse malignancies, including growth, apoptosis, invasion, metastasis, epithelial-mesenchymal transition, and drug resistance (22).

### 3.1 Mutations in the pathway

The p110 subunit, which is encoded by PIK3CA, has been extensively researched in thyroid cancer and is frequently mutated in various cancers. Within the Class I family of PI3K, PIK3CA is the focus of many studies (23). The prevalence of PIK3CA mutations differs among various types of thyroid cancer. Reports indicate a frequency of 3% in poorly differentiated thyroid carcinoma (PDTC), 14% in well-differentiated papillary thyroid carcinoma (WD-PTC) and 100% in anaplastic thyroid carcinoma (ATC). Studies have shown that in advanced iodine-refractory thyroid cancer patients, PIK3CA mutations are more commonly found, especially in those who develop metastasis or experience recurrence. It is worth noting that thyroid cancer patients with mutations in PIK3CA or AKT1 almost always also have *BRAF* mutations (24), indicating an interaction between the *RAS/BRAF/MAPK* pathway and the PI3K/AKT pathway in driving thyroid cancer development. Moreover, García B et al. demonstrated that insulin-like growth factor (IGF-1) inhibits the expression of NIS *mRNA* induced by TSH and Forskolin, which leads to a reduction in iodine uptake. This inhibitory effect can be attenuated by inhibiting PI3K (25). Therefore, PI3K appears to regulate the expression of NIS and plays a pivotal role in the function of iodine uptake in thyroid cells. Moreover, as discovered by Hou P, Kogai T et al., the blockade of the PI3K pathway notably amplifies the absorption of radioactive iodine by tumourous thyroid cells (26, 27). These findings reinforce the notion that PI3K may have an impact on the development of RAIR-DTC. Phosphorylated p-AktSer473, a signaling molecule downstream of this pathway, is modulated by mTORC2, a protein complex comprising mTOR. Overstimulation of mTORC2 results in reduced expression of the *SLC5A5* gene that encodes NIS *mRNA*, whereas phosphorylated p-AktSer473 strongly influences the metastasis of cells in thyroid tumours (28). The mTORC2-p-AktSer473 pathway is extensively expressed in tumour cells having the *BRAF^V600E^* gene, proposing exertion of control by *BRAF^V600E^* over specific functions of this pathway. In conclusion, aberrant activation of the Akt family integrates multiple signaling pathways to promote distant metastasis in thyroid tumour cells and reduce the expression of NIS proteins. The PI3K/AKT pathway plays a crucial role in thyroid tumour progression and is a potential target for therapeutic intervention.

### 3.2 Negative regulator of the pathway: *PTEN* induces RAIR-DTC occurrence

The phosphatase and tensin homologue *(PTEN*), situated on chromosome 10, acts as a gene that suppresses tumours and plays a role in the growth of follicular thyroid tumours or inactive thyroid cancer when its expression is changed or absent. To clarify the role of *PTEN* in thyroid disease, Paes JE et al. conducted an experiment by knocking out *PTEN* in mice thyroid cells. Their findings showed a significant increase in the percentage of value-added index ki-67 positivity in thyroid cells that experienced loss of *PTEN*. Additionally, more than two-thirds of female mice developed follicular adenomas. The data suggests that the absence of *PTEN* makes regular thyroid cells vulnerable to neoplastic transformation due to mechanisms linked to cell proliferation (29). In addition, given that the lesions are more prevalent in female mice, it is hypothetically possible that the greater prevalence of thyroid cancer in females than males might be tied to *PTEN* deletion or mutation. Based on the relationship between this negative regulator and NIS in thyroid cells, there is limited evidence reported to date. However, observations have shown that thyroid cancer cells lacking *PTEN* exhibit a significant increase in NIS protein levels primarily localized in the cytoplasm. However, it should be noted that this increase in NIS protein does not lead to a significant increase in iodine uptake by the cells (30). It is possible that the upregulation of *PTEN* is not directly related to the iodine transport function mediated by NIS proteins in thyroid cells. However, since *PTEN* can regulate the PI3K/AKT signaling pathway, and PI3K in this pathway directly affects the iodine uptake capacity of thyroid cells, it is plausible to speculate that there may be a connection between *PTEN* and the iodine uptake capacity of cells. Nonetheless, further studies are needed to confirm this hypothesis. In summary, the dysregulation of the PI3K/AKT signaling pathway is strongly linked with the pathogenesis of RAIR-DTC and could signify a crucial target for therapeutic intervention.

## 4 HMGB1 regulates autophagy

High mobility group box-1(HMGB1), a highly conserved small molecule nuclear protein, is primarily localized in the chromatin of cells. By binding to *DNA*, it exerts regulatory effects on cellular metabolism, proliferation, apoptosis, metastasis, and other biological processes (31, 32). HMGB1 is now recognised to have multiple biological functions both inside and outside the cell, affecting normal cellular activities or contributing to tumourigenesis. Intracellularly, it assists in DNA transcription, leading to a variety of cellular effects. Extracellularly, HMGB1 engages with various receptor pathways, in particular the receptor (RAGE), which binds tightly to HMGB1 and promotes the production of inflammatory mediators and tumour necrosis factor. Ultimately, this RAGE-HMGB1 pathway contributes to the development and metastasis of tumour cells (33, 34)..

The abundant presence of HMGB1 has been detected in thyroid carcinoma cells but not in benign thyroid nodules or adenomas, indicating its involvement in the onset and progression of thyroid cancer. Research has shown that the downregulation of HMGB1 in papillary thyroid carcinoma cells reduces their invasive and metastatic properties. Moreover, HMGB1 functions as a positive regulator of cellular autophagy, a process that has both advantageous and unfavourable impacts. Although autophagy can remove damaged or ageing proteins and organelles and recycle useful materials from deteriorated cells, it may also prevent cellular uptake of iodine. Autophagy induction using Hanks’ solution has been demonstrated to lower NIS expression and iodide uptake in HMGB1-expressing cells. Conversely, when autophagy inhibitors were used, the opposite effect was observed. Furthermore, autophagy induction results in the production of a substantial amount of reactive oxygen species (ROS), a critical signalling molecule in cellular life and death processes (35), mainly derived from the mitochondrial electron transport chain (mETC). Tang D et al. showed that HMGB1’s translocation is facilitated by ROS, extending its autophagic effect through the use of different mETC inhibitors (36).

Experimental data from the group led by Chai W also confirms the involvement of ROS in autophagy mediated by HMGB1. Additionally, under certain circumstances, ROS influences the AMP-activated protein kinase (AMPK) pathway, which is a kinase that targets serine/threonine proteins, as well as its protein target, mTOR. Hyperphosphorylation of AMPK and degradation or degeneration of NIS proteins were observed in solutions that successfully induced autophagy without ROS scavengers. In contrast, opposite outcomes were noted in the control group. Additionally, Tavares C et al. demonstrated that mTOR is associated with capsule invasion and distant metastasis in thyroid tumor cells and exhibits a negative correlation with NIS protein expression (37). Thus, it is feasible that NIS degradation, facilitated by HMGB1 via autophagy, transpires through the ROS/AMPK/mTOR pathway.

Given the relationship between HMGB1 and tumour cells described above, this mechanism of HMGB1-mediated autophagy may contribute to the reduced sensitivity of RAIR-DTC to radioiodine therapy. Exploring the development of novel targeted drugs with definitive therapeutic effects against HMGB1 may be a promising research direction for the treatment of RAIR-DTC.

## 5 Altered sonic hedgehog-GLI1 signaling pathway

The *Sonic Hedgehog-GLI1* signaling pathway is a highly conserved pathway that plays a critical role in embryonic development, cell proliferation, differentiation, and stem cell maintenance (38). Recent studies have revealed that *GLI1*, a downstream molecule of this pathway, is implicated in thyroid carcinogenesis. It influences the expression of NIS in thyroid cells and regulates the crosstalk between tumour cells and the surrounding stroma, thereby promoting the migration and invasion of thyroid cancer cells (39).

### 5.1 NIS expression and localisation influenced by downstream signaling molecule GLI1

Alessia and colleagues found that there was a negative expression of GLI1 in normal thyroid tissues, but a positive expression in thyroid tumors (39). Similar findings were reported by Oh JM et al., indicating a potential link between GLI1 and thyroid cancer. To explore the correlation between GLI1 and NIS expression in thyroid cells, Oh JM et al. conducted a knockdown of GLI1 and observed a nearly 1.49-fold increase in NIS protein expression compared to the control group. Furthermore, GLI1 also affected the localization of NIS. Results from a western blot analysis showed that in cells lacking GLI1, a considerable portion of NIS translocated from the cytoplasm to the cell membrane, which improved the efficiency of iodine uptake by thyroid cells. This provided convincing evidence that GLI1 knockdown could restore the affinity of thyroid cells for radioactive iodine (RAI). Undoubtedly, it is contended that this restoration of affinity was linked to increased thyroid-specific proteins and transcription factors following the inhibition of GLI1. It is worth mentioning that this impact on promoting NIS localization is also observed in the presence of the *BRAF^V600E^* gene (40). In conclusion, GLI1 is associated with the initiation and progression of thyroid cancer, particularly in the context of RAIR-DTC, a subtype of thyroid cancer.

### 5.2 Interaction of extrapathway factors with the SHH Pathway

Two mutated forms, *HRAS^GV12^* and *BRAF^V600E^*, have been identified as stimulators of the SHH pathway, leading to increased GLI1 activity. The use of MEK inhibitors has also been found to significantly reduce GLI1 *RNA* expression. Furthermore, the interstitial space of thyroid cancer cells can generate a paracrine factor that synergistically affects the SHH pathway, predominantly affecting the invasive behaviour of thyroid cancer cells. Currently, no data exists to indicate that this secretory factor contributes to the proliferation process of thyroid cancer cells. It is evident that without timely intervention, this harmful cycle persists and worsens the progression and deterioration of thyroid cancer (39). In summary, activating the SHH pathway through mutations in the *RAS/BRAF/MEK* pathway and the presence of paracrine factors in the interstitial space of thyroid cancer cells can enhance GLI1 expression and activity. Consequently, the pathway controls the iodine uptake by thyroid cells and the abnormal biological behavior of thyroid tumor cells. These findings reiterate the importance of considering potential synergistic interactions of multiple pathways in the development of RAIR-DTC.

## 6 Discussion

Iodine-refractory differentiated thyroid cancer presents significant challenges due to its difficulty in early diagnosis and resistance to multiple treatment modalities. Addressing this issue requires a comprehensive understanding of its molecular mechanisms, identification of critical mutated genes or signaling pathways, restoration of cellular differentiation capabilities, and reestablishment of sodium/iodine symporter expression activity. These measures are essential for effective treatment. Further investigation into targeted drugs with accurate efficacy is needed to determine the best timing for treatment. For patients with intricate medical conditions, a thorough analysis of pathogenesis using cross-talk with several mutational pathways is imperative. However, the pathogenesis and treatment of RAIR-DTC remain incompletely understood, with researchers worldwide actively investigating potential solutions. Additionally, toxicity and side effects linked to current targeted drugs must be carefully evaluated and addressed. We believe that forthcoming clinical research will clarify the pathogenesis of RAIR-DTC and identify targeted drugs appropriate for most patients. Concurrently, the management plan for patients with RAIR-DTC will gradually become clearer and enhanced.

## Author contributions

SZ: Writing – original draft. YZu: Writing – original draft. YZo: Writing – review & editing. GW: Writing – review & editing.
