# Supplementary material for: Pathogenesis and signaling pathways related to iodine-refractory differentiated thyroid cancer
Source: Front Endocrinol (Lausanne). 2024 Jan 19;14:1320044. doi: 10.3389/fendo.2023.1320044 (PMC10836590; doi:10.3389/fendo.2023.1320044)
Supplement: Supplementary file 1 [file Image_1.pdf]

Supplementary Material

Pathogenesis and signalling pathways related to iodine-refractory differentiated thyroid cancer

Simeng Zhao[1†], Yuejia Zhao[2†], Yongfu Zhao*, Guangzhi Wang*

*** Correspondence:** Guangzhi Wang, wanggz@dmu.edu.cn or Yongfu Zhao, dl.zyf67@163.com

1 Supplementary Figures

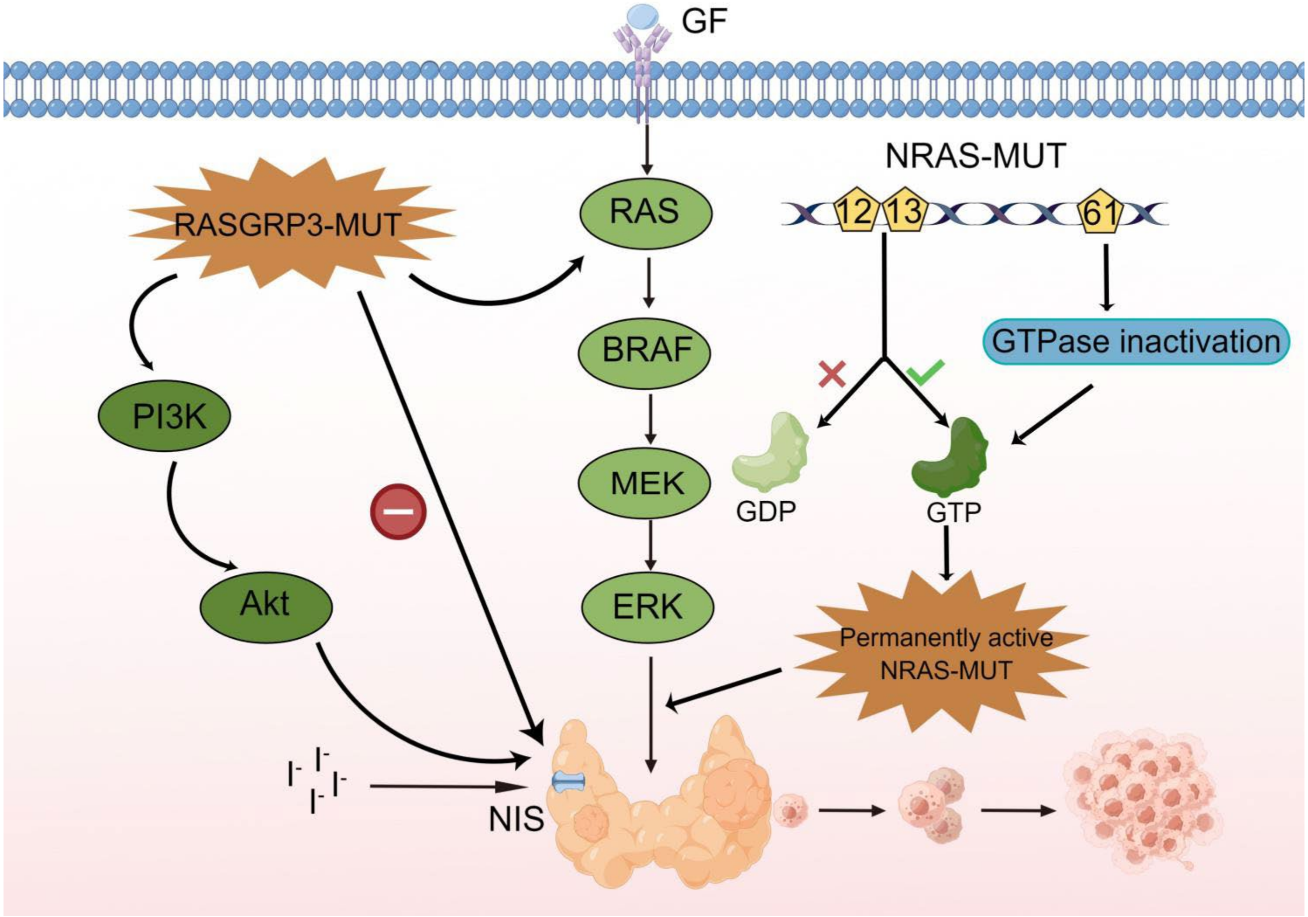

Supplementary Figure 1. The mechanism of action by which *RAS* mutations promote the occurrence and invasion of thyroid tumor cells and inhibit the iodine uptake ability of NIS. One of the mutated variations of *RAS*, known as *NRAS*, is frequently identified in the advancement of thyroid cancer. It encompasses three codons that are prone to mutation, namely 12, 13, and 61. Codons 12 and 13 perform the function of binding to inactive GDP to govern various regular cellular operations. Yet, when their mutation takes place, GDP is released that binds to active GTP, thereby

governing the growth of cancerous cells. Codon 61 permanently deactivates guanosine triphosphatase through its own catalytic effect and subsequently binds to active GTP leading to an increase in thyroid cancer incidence. On the contrary, *RAS*GRP3, a different mutated form of *RAS*, may hinder iodine uptake and promote thyroid cancer in NIS through the PI3K/AKt pathway.

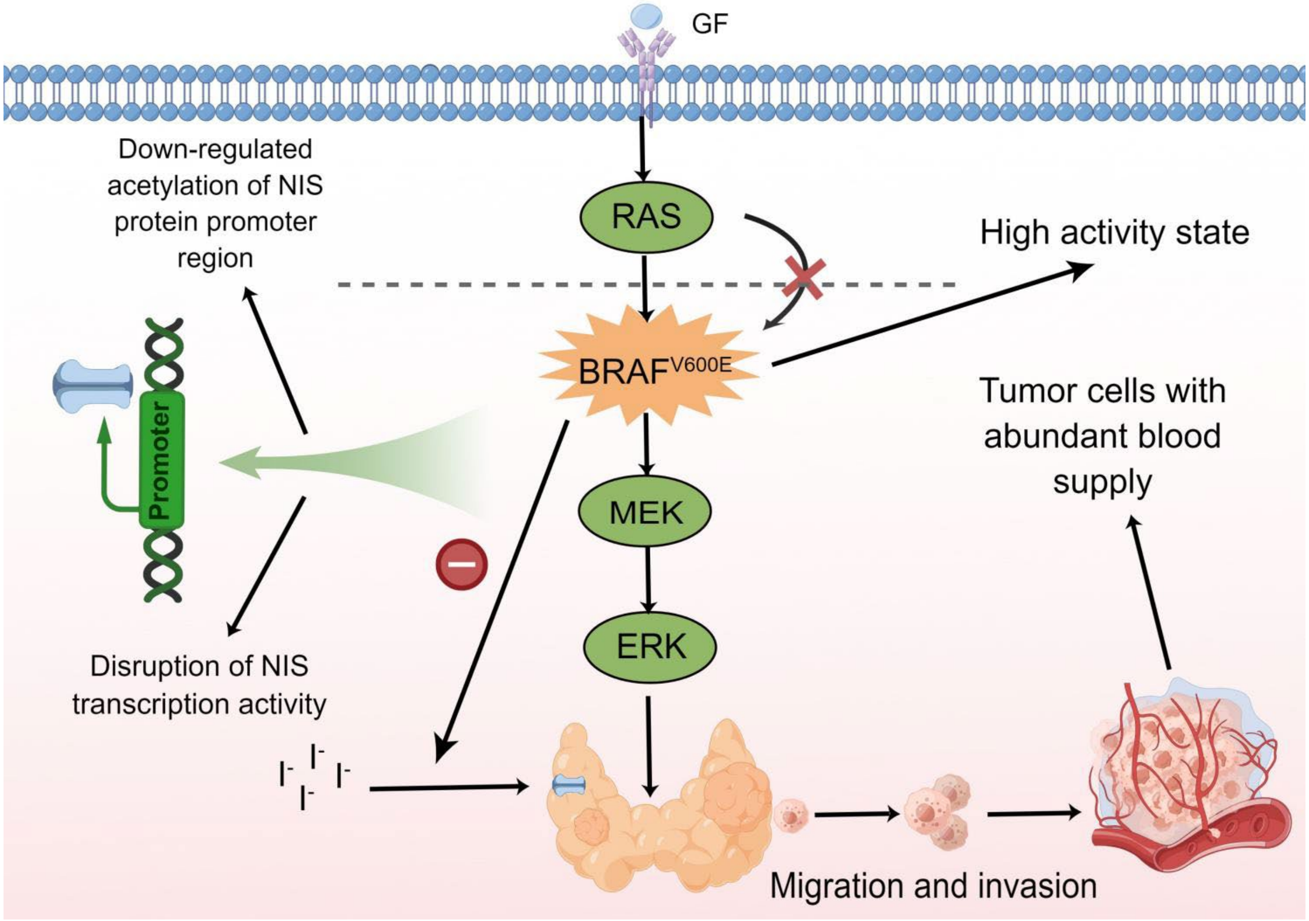

Supplementary Figure 2. *BRAF^{V600E}* is a significant contributor to the onset of RAIR-DTC due to its independence and high level of activity. It can activate downstream signaling pathways autonomously, supplying a significant amount of blood to thyroid tumor cells without relying on upstream RAS stimulation. This ability leads to rapid metastasis and invasion. BRAFV600E inhibits

NIS expression by selectively down-regulating histone acetylation in pivotal regions of the NIS gene promoter in thyroid cells, consequently averting iodine absorption.

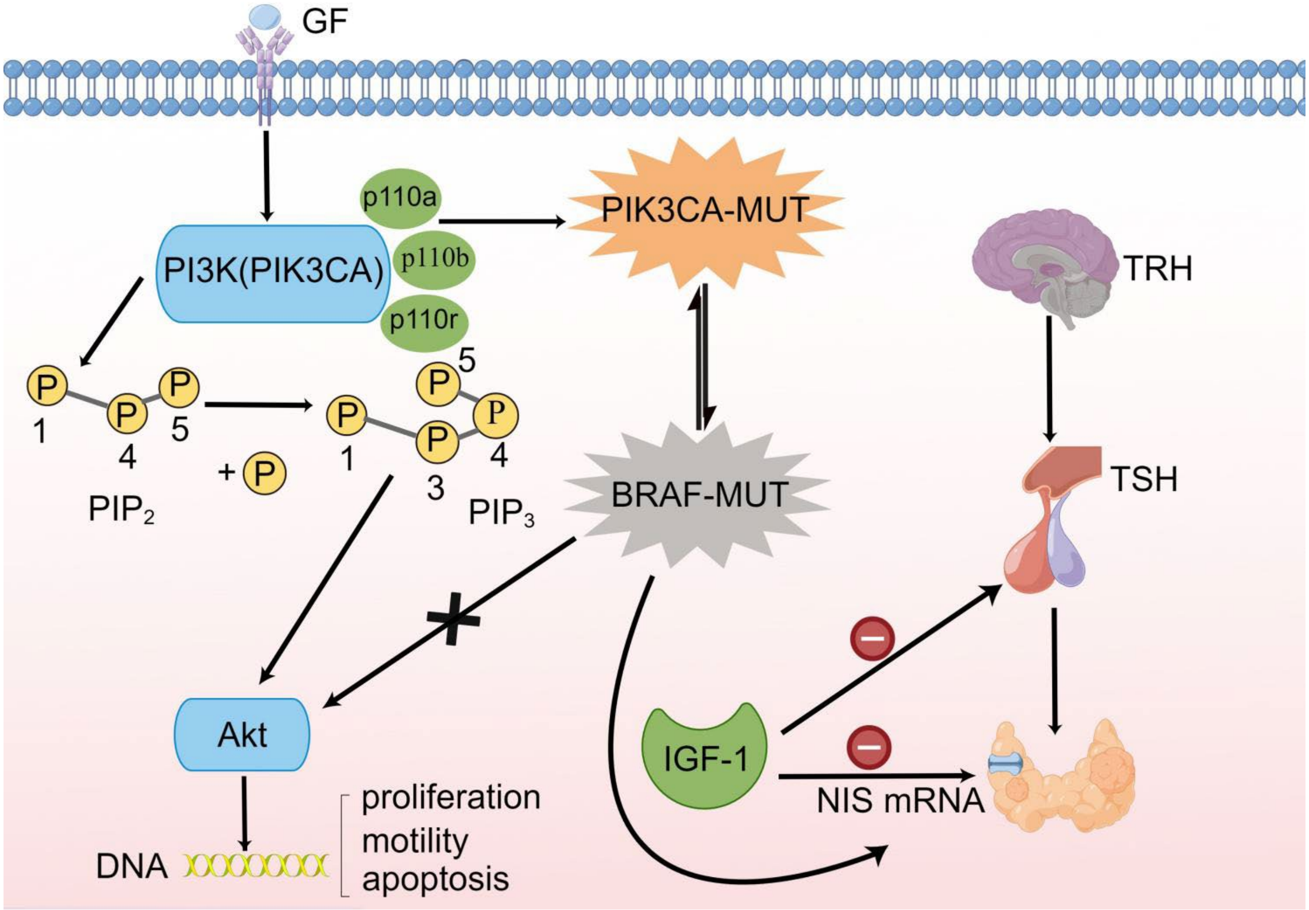

Supplementary Figure 3. PIK3CA, *BRAF* mutations and IGF-1 are interconnected and jointly promote the occurrence of RAIR-DTC. The PI3K family regulates the normal biological behavior of the nucleus by phosphorylating inositol bisphosphate to form inositol triphosphate. However, abnormal activation of the catalytic subunit p110 of PI3K to form mutations (PIK3CA-MUT) inhibits the above pathway and contributes to the development of thyroid cancer. *BRAF* mutations are closely

linked to PIK3-MUT and can promote the pathogenesis of RAIR-DTC by modulating IGF-1's inhibitory effect on TSH and NIS *mRNA*.

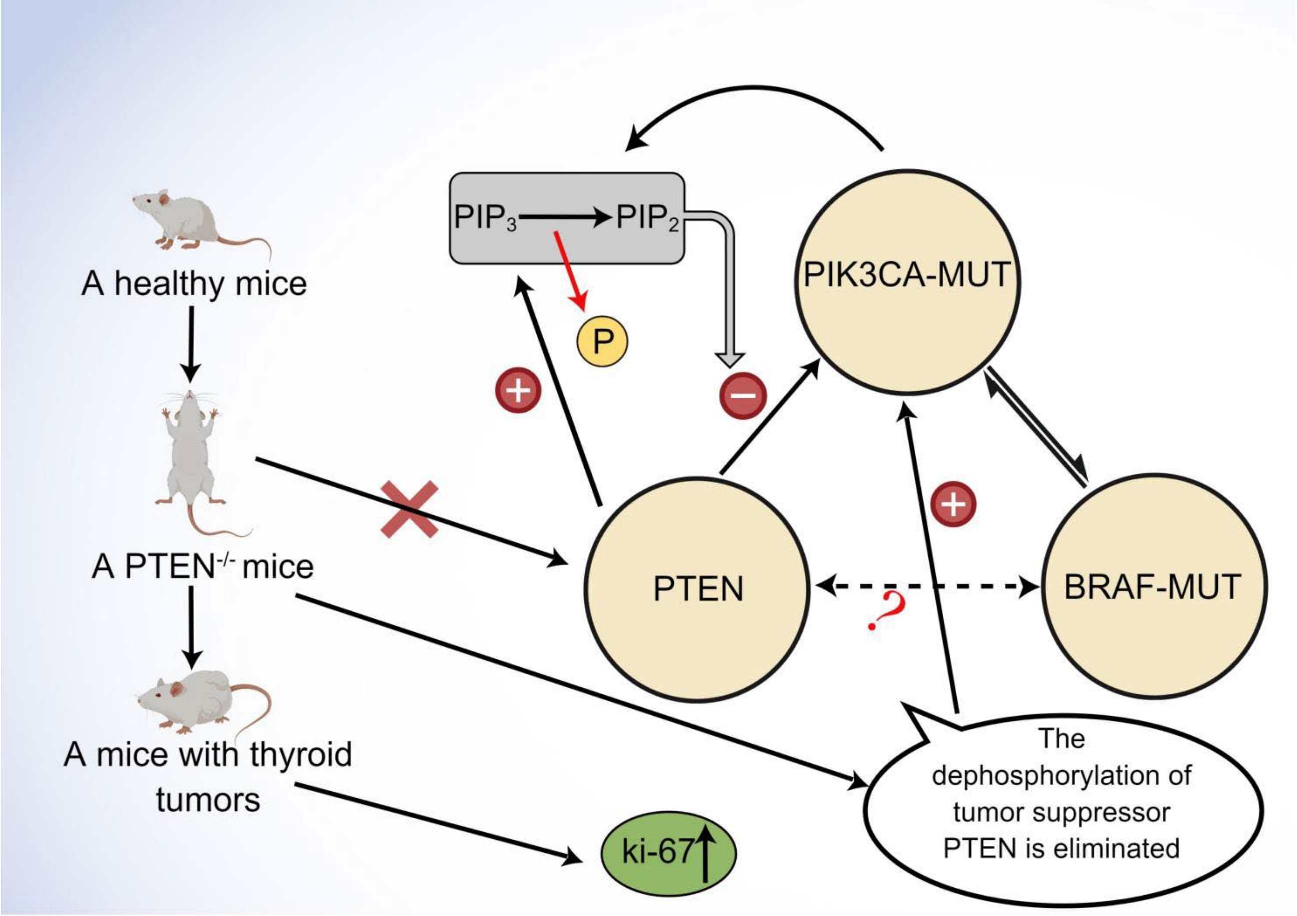

Supplementary Figure 4. *PTEN*, a negative regulatory gene of the PI3K/AKT pathway, enhances the proliferation of thyroid tumour cells. As a tumour suppressor gene, *PTEN*'s inhibition mechanism in the development of thyroid cancer may arise from the dephosphorylation of PIK3CA-MUT. The absence or abnormality of *PTEN* results in a significant increase in the proliferation index ki-67 of

thyroid cells, which does not eliminate further neoplastic transformation. Furthermore, the correlation between *PTEN* and *BRAF* is currently unclear, requiring further research to elucidate.

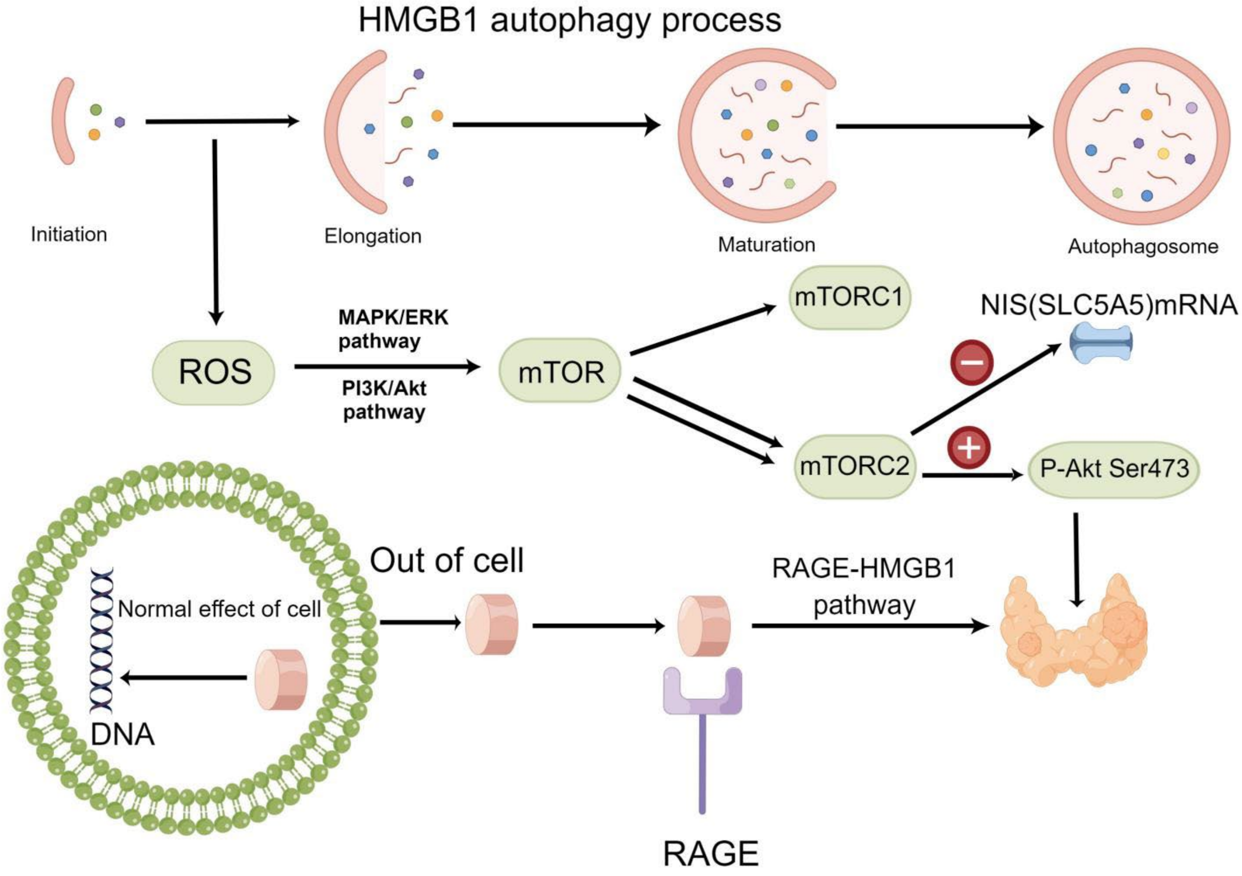

Supplementary Figure 5. ROS, an intermediate product of the HMGB1-mediated autophagy effect, leads to the occurrence of RAIR-DTC. ROS originates from the mitochondrial electron transport chain. HMGB1's mediation of autophagy maintenance is due to positive feedback from its intermediate product, ROS. ROS can stimulate mTOR through two signaling pathways, MAPK/ERK or PI3K/Akt. MTOR is more easily converted to mTORC2 in this form. Abnormal activation of mTORC2 can impede the transcription of *SLC5A5* in NIS, resulting in decreased iodine uptake. On the contrary, an overactivation of mTORC2 triggers excessive activation of phosphorylated P-Akt Ser473, which in turn contributes to the functioning of malignant cells in thyroid tumors. HMGB1, via the RAGE-HMGB1 pathway outside the cell, can precisely partake in the biological conduct of

these malignant cells. Thus, the onset of thyroid cancer may result from the concerted influence of multiple signaling pathways.

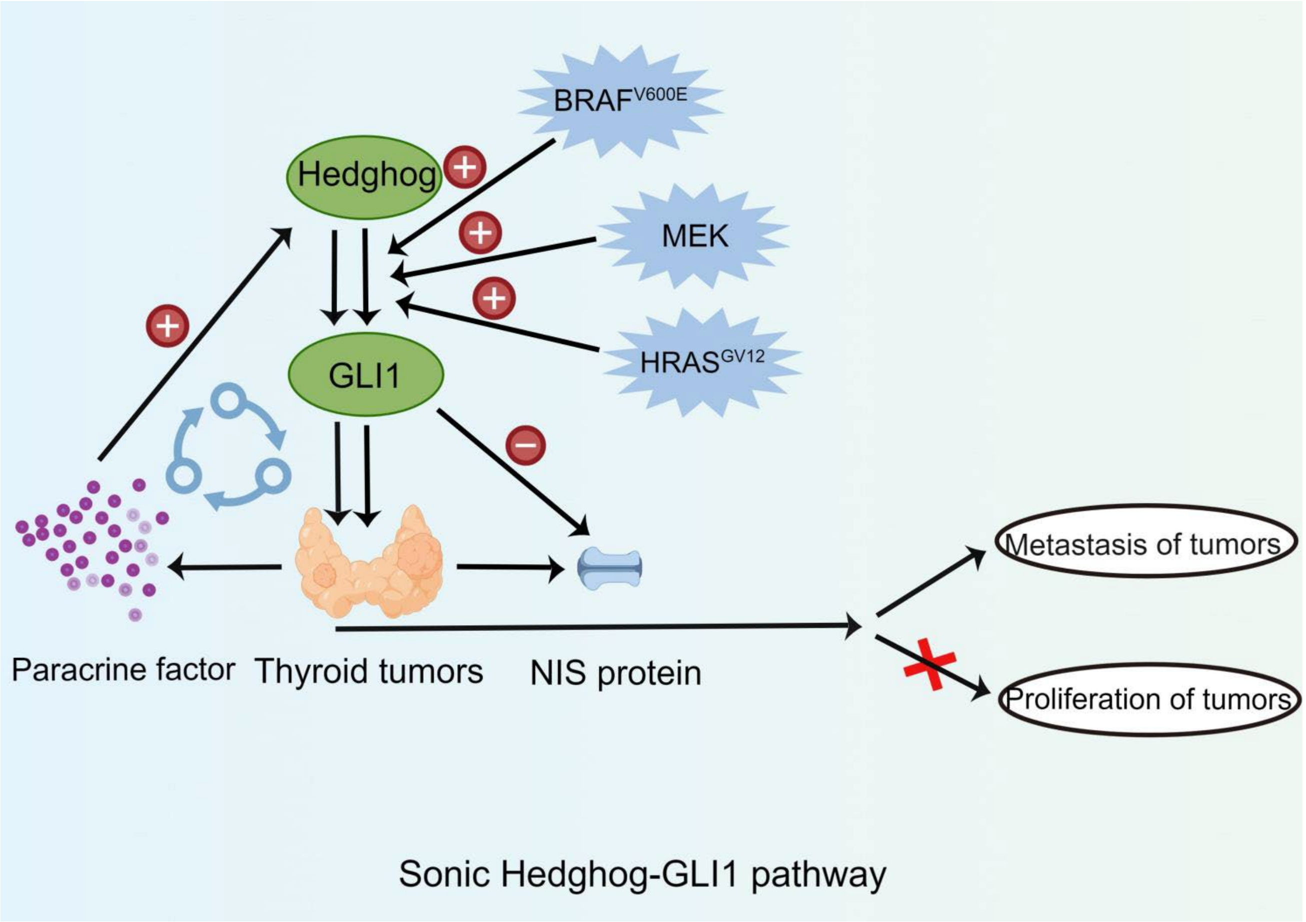

Supplementary Figure 6. Aberrant elements inside and outside of the Sonic Hedgehog-GLI1 pathway result in the pathogenesis of RAIR-DTC. Irregularly activated $BRAF^{V600E}$, MEK, and $HRAS^{GV12}$ have the potential to boost the SHH-GLI1 pathway and hinder the thyroid cells' iodine uptake, leading to the proliferation of cancer cells. The pathway generates a paracrine factor that increases the expression activity of GLI1. GLI1 is generally thought to impede the uptake of iodine by thyroid cells, thus encouraging thyroid tumorigenesis. So this paracrine factor thus hastens the development of a harmful cycle.
